# Prognostic relevance of high pretreatment CA125 levels in primary serous ovarian cancer

**DOI:** 10.3892/mco.2021.2247

**Published:** 2021-02-26

**Authors:** Robert Bachmann, Sara Brucker, Annette Stäbler, Bernhard Krämer, Ruth Ladurner, Alfred Königsrainer, Diethelm Wallwiener, Cornelia Bachmann

Mol Clin Oncol 14:Article no. 8, 2021; DOI: 10.3892/mco.2020.2170

Following the publication of this article, the authors have requested that the first sentence in the Materials and methods section, as it appeared towards the bottom of the left-hand column of the second page, be amended as follows (essentially, the dates have been revised, as indicated in bold): “A total of 136 consecutive patients with primary epithelial ovarian cancer and existing pretreatment CA125 levels .500 U/ml and CA125 <500 U/ml at the time of surgery treated at the department of obstetrics and gynecology from **January 2000 through June 2007** at University Tübingen, Germany were enrolled in the study and retrospectively analyzed.”

The authors regret that this error went unnoticed before the publication of the article, and all authors agree with publishing this Corrigendum; moreover, this change does not affect any of the major conclusions reported in the study. The authors are grateful to the Editor of *Molecular and Clinical Oncology* for allowing them the opportunity to publish this Corrigendum, and apologize to the readership of the Journal for any inconvenience caused.

